# Targeted lipid‐siRNA complexes for Spp1 silencing in Alzheimer's Disease

**DOI:** 10.1002/alz70861_109009

**Published:** 2025-12-23

**Authors:** Adam Abdulrahman, Nora Francini, Alexander Ligocki, Alexander Sorets, Andrew Kjar, Matthew Schrag, Craig Duvall, Ethan S Lippmann

**Affiliations:** ^1^ Vanderbilt University, Nashville, TN USA; ^2^ Vanderbilt University Medical Center, Nashville, TN USA

## Abstract

**Background:**

Alzheimer's disease (AD) is a progressive neurodegenerative disorder where neuroinflammation plays a central role. Border‐associated macrophages (BAMs), which uniquely express the mannose receptor CD206 in the CNS, are increasingly implicated as contributors to this inflammatory response. The protein secreted phosphoprotein 1 (SPP1), encoded by the Spp1 gene, is upregulated in BAMs in AD and exacerbates neuroinflammation. Targeting SPP1 in BAMs represents a promising strategy to investigate, and mitigate, the drivers of neuroinflammation in AD. Small interfering RNA (siRNA) therapeutics offer a precise method to silence disease‐associated genes like Spp1.

**Method:**

To target Spp1 in BAMs, we developed two siRNA‐based approaches: (1) L2‐siRNA: A divalent lipid‐conjugated siRNA. (2) Man3‐siRNA: A tri‐mannose‐conjugated siRNA designed for CD206‐mediated BAM‐specific uptake. In vitro, we assessed the efficacy of gene silencing and macrophage uptake using RAW264.7 and J774A.1 cell lines, as well as primary bone marrow‐derived macrophages, through qPCR and flow cytometry. In vivo, we administered these siRNAs intracerebroventricularly (ICV) to murine models, evaluating biodistribution and gene silencing using confocal microscopy, flow cytometry, and single‐cell RNA sequencing.

**Result:**

L2‐siRNA achieved robust, dose‐dependent gene silencing in vivo, with widespread distribution in the CNS after ICV injection. It demonstrated preferential uptake by BAMs while minimizing off‐target effects in other CNS cells and peripheral organs. Man3‐siRNA showed selective uptake in CD206^hi macrophages in vitro (IC50 ∼12 nM) and localized primarily to perivascular regions in vivo, confirming BAM‐specific targeting. Additionally, mannan competition assays confirmed that man3‐siRNA uptake is CD206‐dependent, as uptake was inhibited by mannan. We also identified a potent siRNA targeting Spp1 through lipofectamine‐mediated screening in RAW264.7 macrophages, and confirmed its activity in the L2‐siRNA format in both RAW264.7 and J774A.1 macrophages, demonstrating robust suppression of Spp1 expression compared to a non‐targeting control.

**Conclusion:**

Both L2‐siRNA and man3‐siRNA effectively localize to BAMs, with man3‐siRNA offering enhanced specificity via CD206 targeting. These approaches show potential for modulating neuroinflammation in AD by selectively targeting BAMs, potentially minimizing systemic side effects. Further studies will investigate the therapeutic impact of Spp1 silencing on AD progression.

